# Prognostic modeling for diffuse midline glioma: development and validation of a risk stratification nomogram using SEER and institutional cohorts

**DOI:** 10.3389/fonc.2025.1644388

**Published:** 2025-10-27

**Authors:** Ge Zhang, Huandi Zhou, Wanyue Han, Lei Lou, Xiaoying Xue

**Affiliations:** ^1^ Department of Radiotherapy, The Second Hospital of Hebei Medical University, Shijiazhuang, Hebei, China; ^2^ Hebei Key Laboratory of Etiology Tracing and Individualized Diagnosis and Treatment for Digestive System Carcinoma, The Second Hospital of Hebei Medical University, Shijiazhuang, Hebei, China; ^3^ Department of Central Laboratory, The Second Hospital of Hebei Medical University, Shijiazhuang, Hebei, China; ^4^ Department of Pathology, The Second Hospital of Hebei Medical University, Shijiazhuang, Hebei, China

**Keywords:** diffuse midline glioma, prognostic nomogram, survival analysis, radiotherapy, chemotherapy

## Abstract

**Background:**

Diffuse midline glioma (DMG) is a rare and highly aggressive central nervous system tumor with limited treatment options and poor survival outcomes. Reliable prognostic models are urgently needed to guide risk-adapted therapy.

**Methods:**

We retrospectively analyzed 409 DMG patients from the SEER database (2018–2021). Independent prognostic factors were identified using multivariate Cox regression analysis. A nomogram was developed to estimate overall survival, and its performance was evaluated using the concordance index (C-index), time-dependent ROC curves, calibration plots. Risk stratification was based on nomogram total scores. Subgroup survival comparisons were conducted using Kaplan–Meier and log-rank tests. External validation was performed using an independent institutional cohort of 22 patients.

**Results:**

An age-dependent anatomical distribution was observed: brainstem tumors predominated in children, while non-brainstem tumors were more common in adults. Multivariate Cox regression identified older age, higher household income, and cerebellar location as independent prognostic factors. These variables were incorporated into a nomogram that demonstrated good discriminative ability and calibration. Based on total risk scores, patients were stratified into high- and low-risk groups with significantly different survival outcomes. Combined chemoradiotherapy significantly improved survival compared to radiotherapy or chemotherapy alone, while chemotherapy alone showed no added benefit. Surgical resection extent was not associated with prognosis. In an external validation cohort of 22 patients, survival was better in the low-risk group than in the high-risk group, although the difference was not statistically significant (P = 0.188).

**Conclusion:**

This study presents the first large-scale, SEER-based nomogram for DMG, offering reliable prognostic stratification and reinforcing the survival benefit of combined chemoradiotherapy. The model’s clinical utility is further supported by real-world institutional validation, underscoring its potential to inform individualized treatment strategies in DMG.

## 1 Introduction

Diffuse midline glioma (DMG), characterized by H3 K27 alterations, is one of the most challenging pediatric malignancies. It predominantly arises in midline structures, including the brainstem, thalamus, and spinal cord (1, 2). Formerly classified as diffuse intrinsic pontine glioma (DIPG) based on anatomical location as a radiographic diagnosis (3, 4), DMG is now recognized by the 2021 WHO Classification as a distinct entity defined by both molecular features—particularly the loss of H3K27me3 trimethylation —and midline location (2, 3, 5). While DIPG and DMG share considerable overlap, they are not identical. Although relatively rare, with an annual incidence of approximately 0.1 per 100,000, DMG accounts for nearly 20% of all pediatric CNS malignancies (4).

Despite advances in molecular understanding, the prognosis remains dismal, with a median overall survival (OS) of 9–11 months (6). Current clinical guidelines diverge substantially. For instance, the National Comprehensive Cancer Network (NCCN 2025) recommends upfront radiotherapy without routine chemotherapy in children (7), whereas European Association for Neuro-Oncology (EANO) suggests radiotherapy with temozolomide (8), and American Society of Clinical Oncology-Society for Neuro-Oncology (ASCO–SNO) jointly released that no standard therapy confers a clear benefit outside clinical trials (9). Moreover, while agents like ONC201 show promise, their efficacy is limited by poor blood–brain barrier (BBB) penetration (10–13). These inconsistencies underscore the urgent need for robust prognostic frameworks to inform treatment decisions.

However, prior studies of DMG prognosis have been limited by small sample sizes, subtype heterogeneity, or lack of real-world validation (14–16). To overcome these limitations, we conducted a large-scale, population-based analysis using the Surveillance, Epidemiology, and End Results database (SEER), which covers ~28% of the U.S. population. A total of 409 DMG patients were identified, enabling the construction of the largest prognostic nomogram for this disease. Key contributions of this study include: (1) establishing the largest single-disease cohort of molecularly defined DMG patients; (2) revealing an age-dependent anatomical distribution of tumors; (3) developing and internally validating a nomogram incorporating clinical and socioeconomic factors; and (4) externally validating this model in an independent institutional cohort.

This integrated approach offers new population-level evidence to support individualized risk stratification and optimize therapeutic strategies for DMG patients.

## 2 Material and methods

### 2.1 Study design and data source

This population-based retrospective cohort study utilized data from SEER 22 Registries, which collectively cover approximately 28% of the U.S. population. Patients diagnosed with diffuse midline glioma (DMG) between 2018 and 2021 were identified using the ICD-O-3 morphology code 9385/3. Although the SEER database labels this code as “Diffuse intrinsic pontine glioma, H3 K27M-mutant,” it was introduced in 2018 following the WHO 2016 CNS classification to represent diffuse midline glioma, H3 K27M-mutant. This terminology lag is common in SEER, as legacy names are retained to ensure compatibility with earlier datasets (e.g., “glioblastoma multiforme, NOS [9440/3]” remains listed despite the removal of “multiforme” in recent WHO editions). Extracted variables included demographic characteristics (age, sex, race, household income), clinical information (primary tumor location, time from diagnosis to treatment), and treatment modalities (extent of surgery, radiotherapy, and chemotherapy).

In addition, an independent institutional cohort comprising 22 pathologically confirmed DMG patients treated with chemoradiotherapy was retrospectively collected for external validation. Clinical and treatment data were extracted following the same variable definitions as the SEER cohort.

### 2.2 Cohort definition and data preprocessing

Patients from the SEER dataset were randomly assigned to a training cohort (70%) and a validation cohort (30%) for nomogram construction and internal validation, respectively. Continuous variables were summarized as mean ± standard deviation (for normally distributed data) or median [interquartile range, IQR] (for non-normally distributed data), and compared using Student’s t-test or Mann–Whitney U test, as appropriate. Categorical variables were presented as frequencies and percentages, with group comparisons performed using Chi-square or Fisher’s exact test. We used the missRanger package in R, which performs multiple imputation based on random forest algorithms.

### 2.3 Model construction and internal validation

Univariate Cox proportional hazards regression was used to identify candidate prognostic factors associated with overall survival (OS), and variables with p < 0.20 were entered into a multivariable Cox model. A bidirectional stepwise selection procedure, guided by the Akaike Information Criterion (AIC), was employed to determine independent predictors (p < 0.05).

Based on the final model, a prognostic nomogram was developed using the rms package in R. Model performance was assessed by discrimination and calibration. Discrimination was evaluated using the concordance index (C-index) and time-dependent receiver operating characteristic (ROC) curves (via the riskRegression package). Calibration was evaluated by plotting the predicted versus observed OS at 6, 12, and 24 months.

### 2.4 Risk stratification and external validation

A total risk score was calculated for each patient in the SEER cohort based on the nomogram, and the optimal cutoff value was determined using the survival and survminer packages. Patients were classified into high- and low-risk groups accordingly. Kaplan–Meier survival analysis and log-rank tests were used to assess differences in survival between risk strata.

For external validation, the institutional cohort (n = 22) was stratified using the SEER-derived cutoff. Kaplan–Meier curves were generated, and log-rank tests were conducted to evaluate the prognostic separation between risk groups. All statistical tests were two-sided, and p < 0.05 was considered statistically significant.

## 3 Results

### 3.1 Patient characteristics

We identified 409 patients diagnosed with DMG from the SEER database. The median overall survival (OS) was 9 months (IQR: 4–16 months), and the median age was 12 years. Female patients accounted for 53.8% of the cohort. The brainstem was the most common tumor location (49.4%). Radiotherapy and chemotherapy were administered to 74.1% and 51.3% of patients, respectively. The training and validation cohort distributions are shown in **Table 1**.

**Table 1:** Baseline characteristics of DMG patients in the training and validation cohorts.

Variables	Total (n = 409)	No (n = 78)	Radio (n = 121)	Chemo (n = 28)	Rad+Chemo (n = 182)	*p*
Year of diagnosis, n (%)						0.026
2018	85 (20.8)	7 (9.0)	29 (24.0)	2 (7.1)	47 (25.8)	
2019	116 (28.4)	23 (29.5)	33 (27.3)	6 (21.4)	54 (29.7)	
2020	90 (22.0)	23 (29.5)	28 (23.1)	7 (25.0)	32 (17.6)	
2021	118 (28.9)	25 (32.1)	31 (25.6)	13 (46.4)	49 (26.9)	
Age, Median (Q1, Q3)	13.00 (7.00, 30.00)	17.00 (5.25, 37.50)	9.00 (5.00, 13.00)	16.50 (8.75, 29.50)	20.00 (9.00, 35.00)	< 0.001
Sex, n (%)						0.445
Female	220 (53.8)	40 (51.3)	71 (58.7)	17 (60.7)	92 (50.5)	
Male	189 (46.2)	38 (48.7)	50 (41.3)	11 (39.3)	90 (49.5)	
Race, n (%)						0.004
Hispanic (All Races)	130 (31.8)	23 (29.5)	41 (33.9)	18 (64.3)	48 (26.4)	
Non-Hispanic White	190 (46.5)	38 (48.7)	51 (42.1)	5 (17.9)	96 (52.7)	
Non-Hispanic Black	50 (12.2)	13 (16.7)	18 (14.9)	3 (10.7)	16 (8.8)	
Other	39 (9.5)	4 (5.1)	11 (9.1)	2 (7.1)	22 (12.1)	
Household income, n (%)						0.055
<100,000	321 (78.5)	68 (87.2)	93 (76.9)	25 (89.3)	135 (74.2)	
≥100,000	88 (21.5)	10 (12.8)	28 (23.1)	3 (10.7)	47 (25.8)	
Laterality, n (%)						0.144
Right	68 (16.6)	12 (15.4)	16 (13.2)	4 (14.3)	36 (19.8)	
Left	67 (16.4)	16 (20.5)	12 (9.9)	5 (17.9)	34 (18.7)	
Others	274 (67.0)	50 (64.1)	93 (76.9)	19 (67.9)	112 (61.5)	
Primary Site, n (%)						< 0.001
Brain stem	202 (49.4)	30 (38.5)	84 (69.4)	10 (35.7)	78 (42.9)	
Cerebellum	17 (4.2)	4 (5.1)	3 (2.5)	2 (7.1)	8 (4.4)	
Other	173 (42.3)	40 (51.3)	29 (24.0)	14 (50.0)	90 (49.5)	
Ventricle	17 (4.2)	4 (5.1)	5 (4.1)	2 (7.1)	6 (3.3)	
Time from diagnosis to treatment, n (%)						< 0.001
≤ 7days	186 (45.5)	19 (24.4)	50 (41.3)	13 (46.4)	104 (57.1)	
>7 days	182 (44.5)	24 (30.8)	70 (57.9)	14 (50.0)	74 (40.7)	
Unknown	41 (10.0)	35 (44.9)	1 (0.8)	1 (3.6)	4 (2.2)	
Extent of surgical resection, n (%)						0.083
No	206 (50.4)	42 (53.8)	68 (56.2)	14 (50.0)	82 (45.1)	
partial	168 (41.1)	27 (34.6)	45 (37.2)	9 (32.1)	87 (47.8)	
Subtotal or total	30 (7.3)	6 (7.7)	7 (5.8)	5 (17.9)	12 (6.6)	
Unknown	5 (1.2)	3 (3.8)	1 (0.8)	0 (0.0)	1 (0.5)	
Chemotherapy, n (%)						< 0.001
Yes	210 (51.3)	0 (0.0)	0 (0.0)	28 (100.0)	182 (100.0)	
No	199 (48.7)	78 (100.0)	121 (100.0)	0 (0.0)	0 (0.0)	
Radiation, n (%)						< 0.001
Yes	303 (74.1)	0 (0.0)	121 (100.0)	0 (0.0)	182 (100.0)	
No	106 (25.9)	78 (100.0)	0 (0.0)	28 (100.0)	0 (0.0)	
Radiation Chemotherapy, n (%)						< 0.001
No	78 (19.1)	78 (100.0)	0 (0.0)	0 (0.0)	0 (0.0)	
Radiation	121 (29.6)	0 (0.0)	121 (100.0)	0 (0.0)	0 (0.0)	
Chemotherapy	28 (6.8)	0 (0.0)	0 (0.0)	28 (100.0)	0 (0.0)	
Radio+Chemotherapy	182 (44.5)	0 (0.0)	0 (0.0)	0 (0.0)	182 (100.0)	

### 3.2 Patterns of incidence by age and tumor location

**Figure 1:**
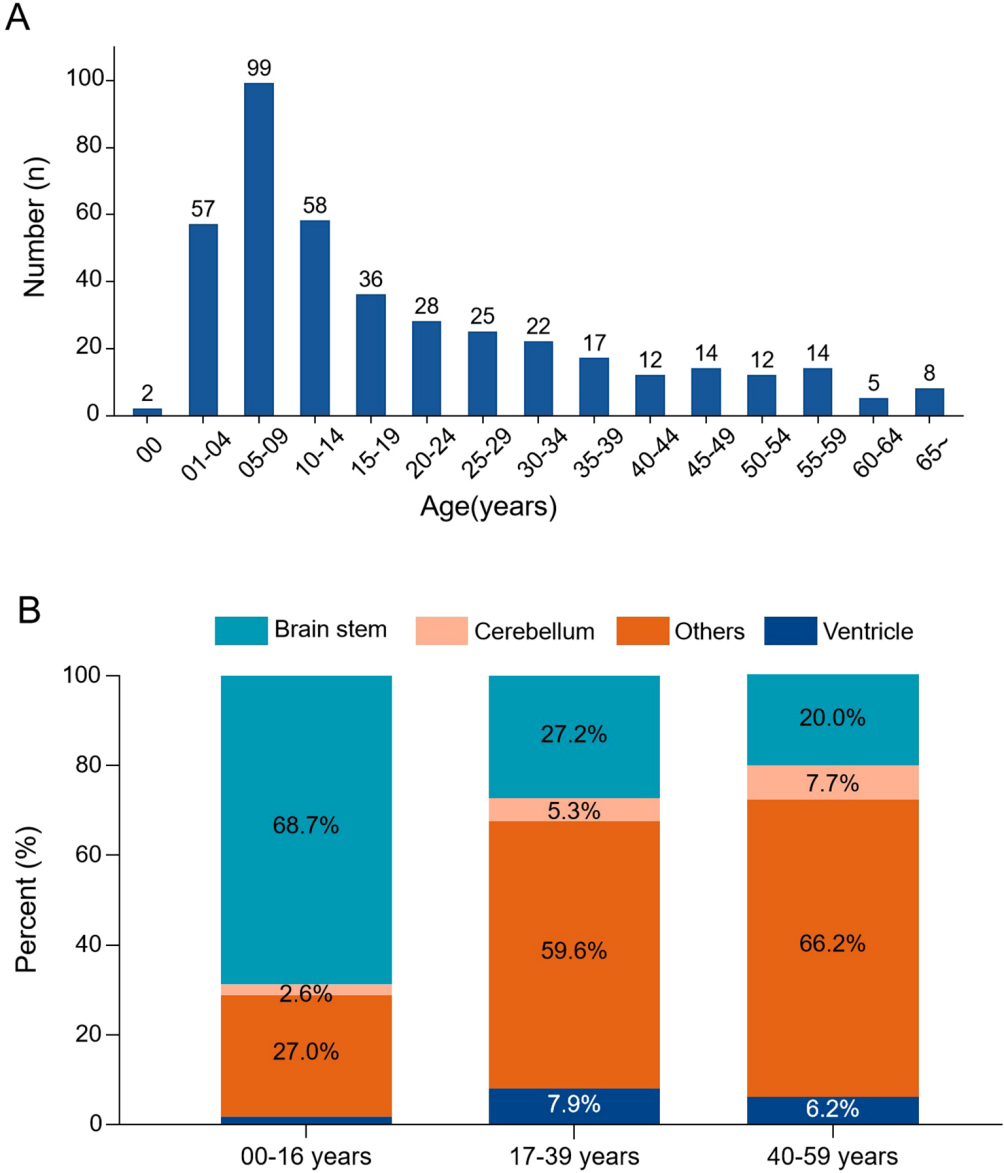
Age distribution and tumor location patterns in DMG. **(A)** Age-specific incidence histogram showing the number of cases across age groups. **(B)** Stacked bar chart illustrating tumor location distribution stratified by age groups. Brainstem tumors predominated in pediatric patients, while non-brainstem locations were more frequent in adults.

The incidence of DMG reached its peak in children aged 5 to 9 years old and gradually decreased thereafter. There was a significant association between age and tumor location (χ²=24.6, p<0.001): brainstem tumors were predominant in patients under 14 years (70.8%), whereas non-brainstem tumors were more common in adults and the elderly (**Figure 1**).

### 3.3 Survival analyses

**Figure 2:**
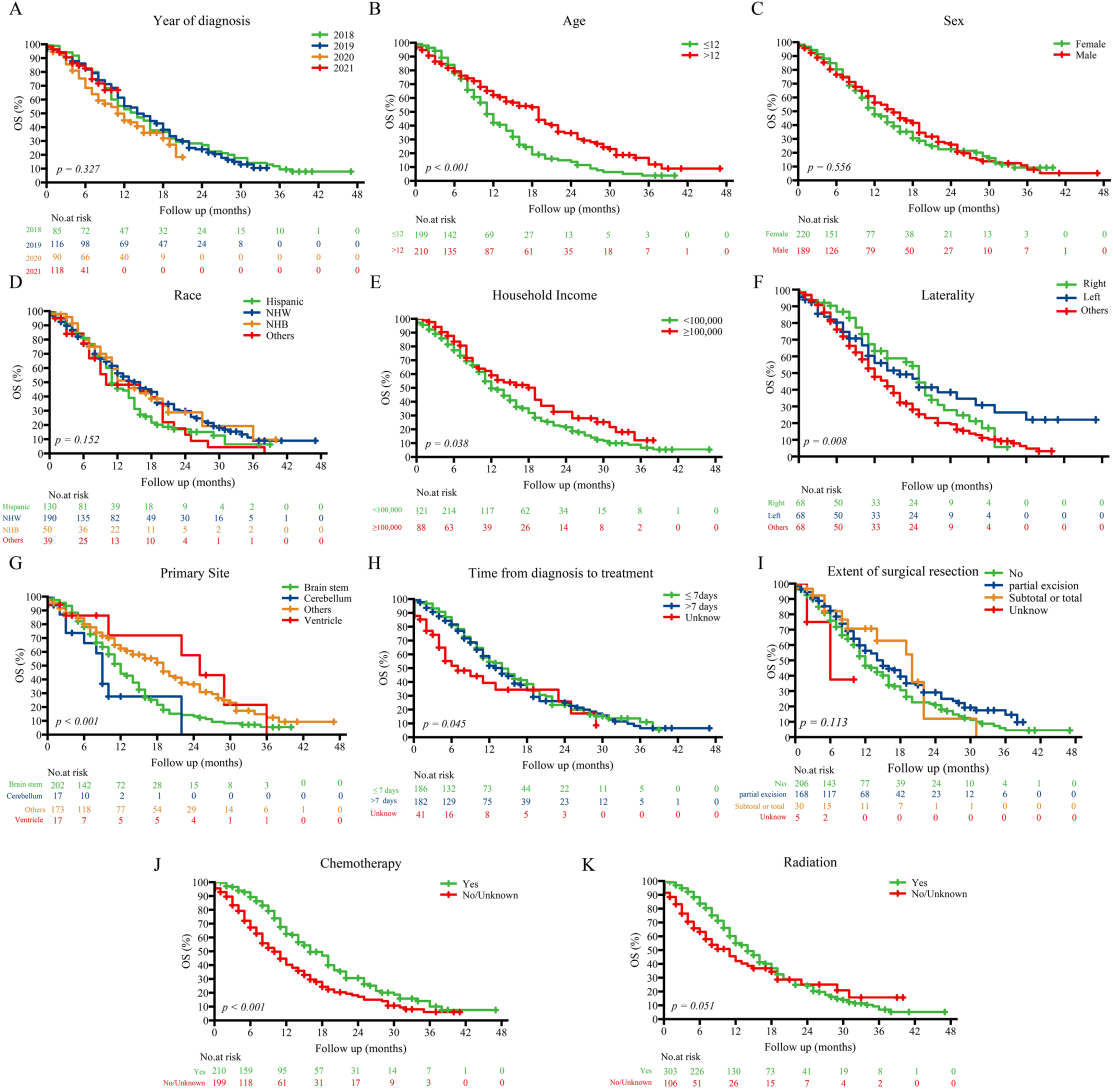
Kaplan–Meier survival curves stratified by prognostic factors in the entire cohort.Kaplan–Meier curves showing overall survival differences across subgroups defined by key prognostic variables: **(A)** Year of diagnosis; **(B)** Age; **(C)** Sex; **(D)** Race; **(E)** Household income; **(F)** Laterality; **(G)** Primary site; **(H)** Time from diagnosis to treatment; **(I)** Extent of surgical resection; **(J)** Chemotherapy; **(K)** Radiation. NHW, Non-Hispanic White; NHB, Non-Hispanic Black.

Kaplan–Meier analysis (**Figure 2**) demonstrated that older age (>12 years; p < 0.001), non-brainstem tumor location (p < 0.001), receipt of chemotherapy (p < 0.001), higher household income (≥$100,000; p = 0.038), and shorter diagnosis-to-treatment intervals (p = 0.045) were significantly associated with improved OS. Sex, race, and extent of surgical resection showed no significant correlation with survival (all p > 0.05).

### 3.4 Cox regression analyses and development of nomogram

Univariate Cox regression analysis (**Table 2**) showed protection factors, including age >12 years (HR = 0.565, 95%CI 0.416-0.769; p<0.001), male sex (HR = 0.724, 95%CI 0.535-0.981; p=0.037), non-Hispanic White ethnicity (HR = 0.678, 95%CI 0.476-0.965; p=0.031), and higher income (≥$100,000; HR = 0.661, 95%CI 0.452-0.968; p=0.033). Conversely, the lack of chemotherapy (HR = 1.814, 95%CI 1.338-2.458; p<0.001) or radiotherapy therapy (HR = 1.608, 95%CI 1.129-2.289; p=0.008) independently predicted poorer results.

**Table 2:** Univariate Cox regression analysis in the training cohorts.

Variable	Estimate	STD.error	Statistic	HR (95%CI)	*p*
Year of diagnosis					
2018	0.000			reference	
2019	-0.018	0.191	-0.093	0.982(0.676,1.428)	*0.926*
2020	0.371	0.224	1.660	1.449(0.935,2.246)	*0.097*
2021	0.388	0.309	1.256	1.474(0.804,2.702)	*0.209*
Age					
​≤12	0.000			reference	
​>12	-0.571	0.157	-3.637	0.565(0.416,0.769)	***<0.001***
Sex					
Female	0.000			reference	
Male	-0.323	0.155	-2.084	0.724(0.535,0.981)	***0.037***
Race					
Hispanic (All Races)	0.000			reference	
Non-Hispanic White	-0.389	0.180	-2.160	0.678(0.476,0.965)	***0.031***
Non-Hispanic Black	-0.056	0.260	-0.215	0.946(0.569,1.573)	*0.830*
Other	-0.040	0.288	-0.138	0.961(0.546,1.690)	*0.890*
Household income ($)					
<100,000	0.000			reference	
≥100,000	-0.414	0.194	-2.130	0.661(0.452,0.968)	***0.033***
Laterality					
Right-origin of primary	0.000			reference	
Left-origin of primary	-0.016	0.280	-0.058	0.984(0.568,1.703)	*0.954*
Others	0.370	0.214	1.731	1.448(0.952,2.203)	*0.083*
Primary Site					
Brainstem	0.000			reference	
Cerebellum	0.597	0.369	1.616	1.817(0.881,3.748)	*0.106*
Other	-0.487	0.164	-2.968	0.614(0.445,0.848)	***0.003***
Ventricle	-0.452	0.462	-0.980	0.636(0.257,1.573)	*0.327*
Time from diagnosis to treatment					
≤ 7days	0.000			reference	
>7 day	0.011	0.162	0.070	1.011(0.737,1.389)	*0.944*
Unknown	0.568	0.256	2.219	1.765(1.068,2.914)	*0.027*
Extent of surgical resection					
No	0.000			reference	
Partial excision	-0.274	0.158	-1.733	0.760(0.558,1.036)	*0.083*
Subtotal or total excision	-0.022	0.370	-0.059	0.978(0.474,2.020)	*0.953*
Unknown	0.366	1.009	0.362	1.441(0.199,10.419)	*0.717*
Radiation Chemotherapy					
No	0.000			reference	
Radiation	-0.238	0.207	-1.149	0.788(0.525,1.183)	*0.251*
Chemotherapy	-0.516	0.529	-0.976	0.597(0.212,1.683)	*0.329*
Radio+Chemotherapy	-0.767	0.206	-3.718	0.464(0.310,0.696)	***<0.001***

Bold values indicate statistically significant results (*p* < 0.05).

**Figure 3:**
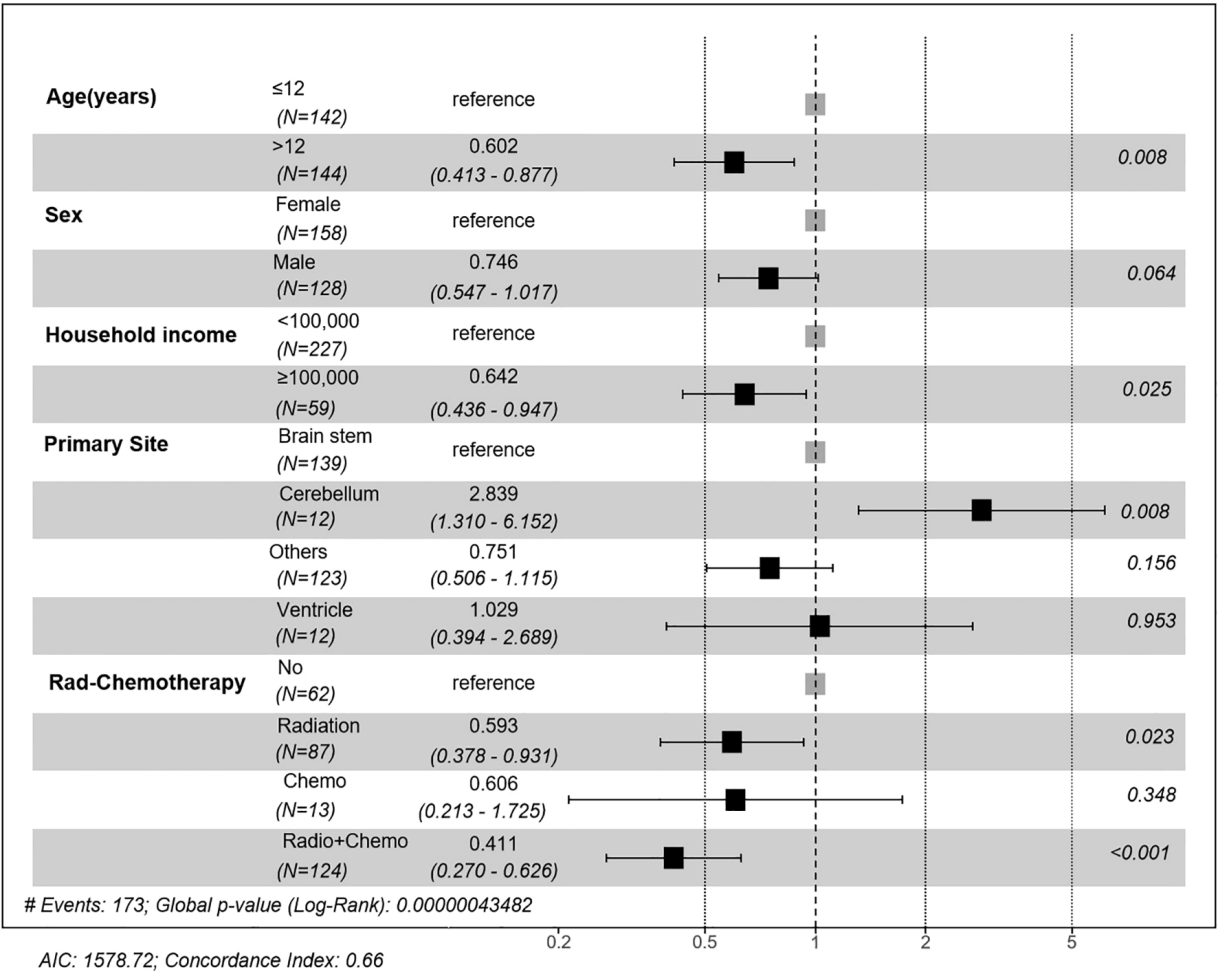
Forest plot of multivariate Cox regression analysis of prognostic factors. Hazard ratios (HR) and 95% confidence intervals (CI) are presented for age, sex, household income, primary site, and treatment modality. Significant prognostic factors included age >12 years, household income ≥$100,000, tumor location, and receiving radiotherapy or combined chemoradiotherapy.

Multivariate Cox regression (**Figure 3**) confirmed that age >12 years (HR = 0.602, 95%CI 0.413-0.877; p=0.0083), household income ≥$100,000 (HR = 0.642, 95%CI 0.436-0.947; p=0.0254), and cerebellum tumors location (vs. brainstem: HR = 2.839, 95%CI 1.310-6.152; p=0.0082) as independent prognostic factors. Therapeutically, radiotherapy alone (HR = 0.593, 95%CI 0.378-0.931; p=0.0232) and combined chemoradiotherapy (HR = 0.411, 95%CI 0.270-0.626; p<0.001) significantly improved survival compared with no/unknown treatment, whereas chemotherapy alone showed no benefit (HR = 0.606, 95%CI 0.270-1.725; p=0.3481).

**Figure 4:**
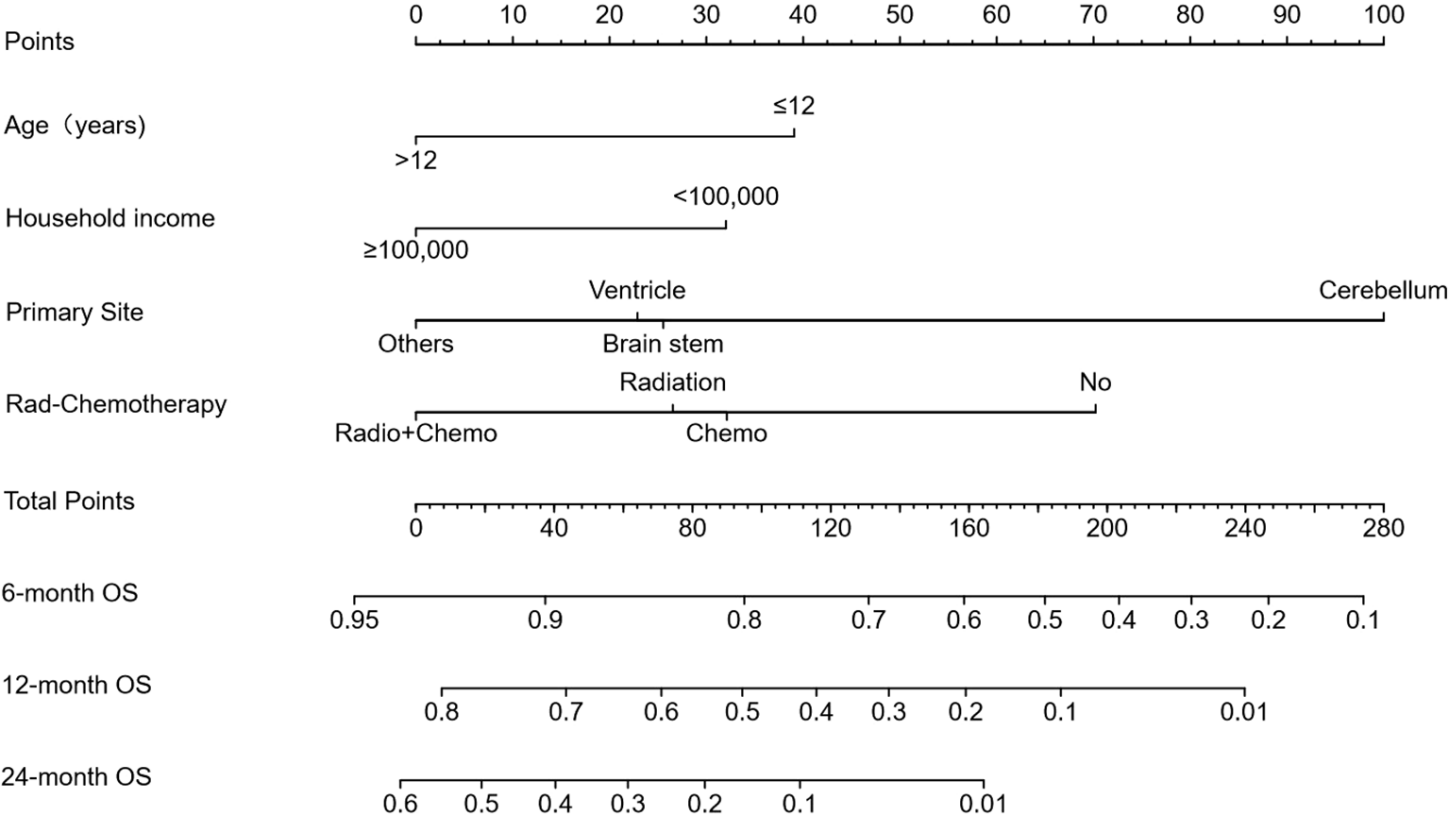
Prognostic nomogram for predicting 6-, 12-, and 24-month overall survival (OS). The nomogram was constructed based on age, household income, tumor location, and treatment modality. Points are assigned for each variable, and total points correspond to predicted survival probabilities at specified timepoints.

Based on these independent predictors, we developed a nomogram predicting OS at 6, 12, and 24 months (**Figure 4**). Younger age (≤12 years), lower income, cerebellum location, and lack of treatment (radiotherapy or chemotherapy) corresponded to higher nomogram scores, indicating poorer prognosis.

### 3.5 Nomogram evaluation and risk stratification

**Figure 5:**
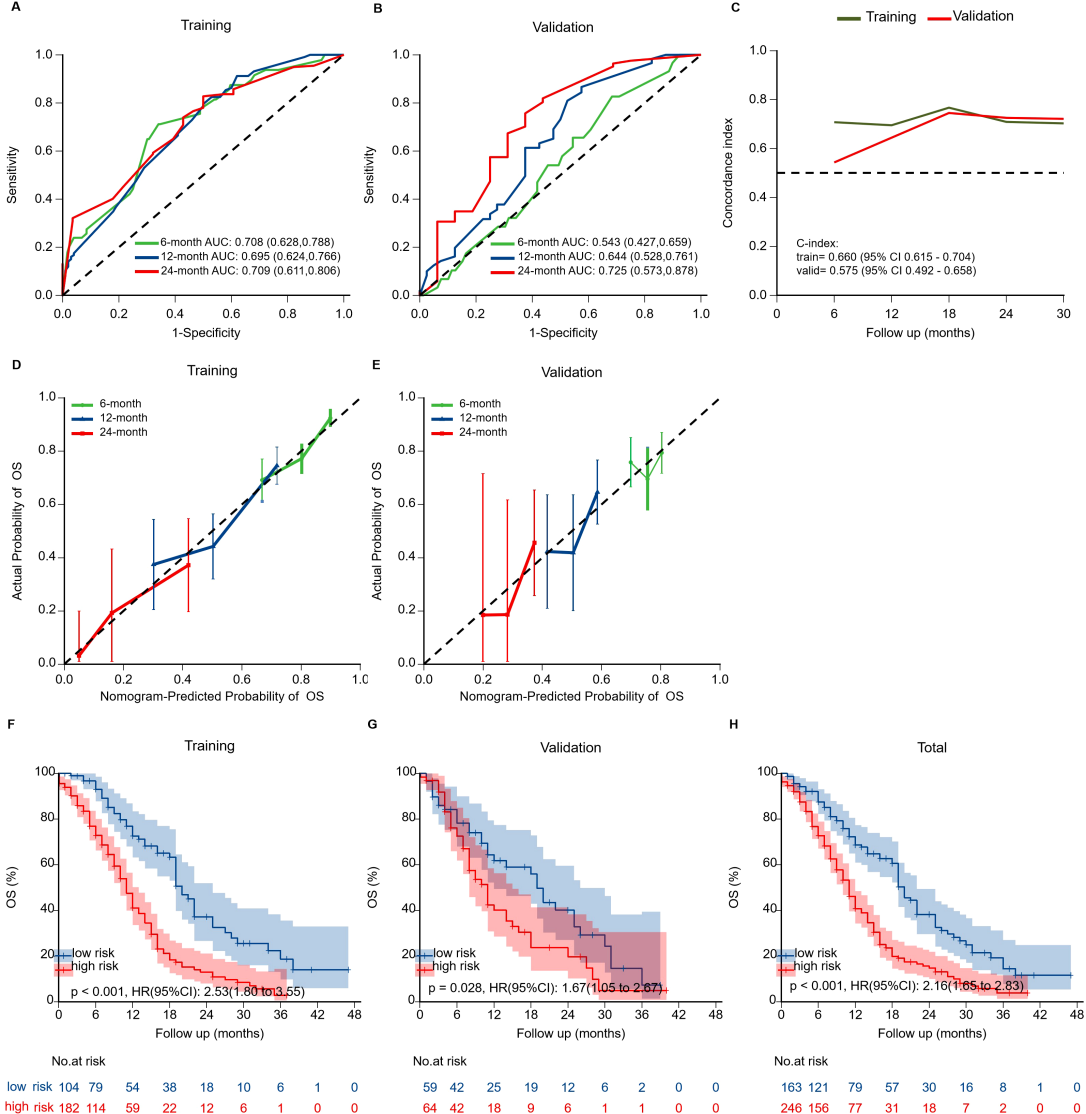
Validation of the prognostic nomogram. **(A, B)** Time-dependent ROC curves evaluating nomogram discrimination in training and validation cohorts. **(C)** Time-dependent concordance index (C-index) curves for both cohorts. Overall C-index was 0.660 (95% CI: 0.615–0.704) in the training cohort and 0.575 (95% CI: 0.492–0.658) in the validation cohort. **(D, E)** Calibration curves for 6-, 12-, and 24-month OS showing agreement between predicted and observed survival. **(F-H)** Kaplan-Meier survival curves stratified by risk groups in training, validation, and overall cohorts. High-risk patients showed significantly worse OS than low-risk patients in all cohorts.

The nomogram exhibited moderate discrimination in both training (6-month AUC = 0.708; C-index=0.660) and validation cohorts (24-month AUC = 0.725; C-index=0.575) (**Figure 5**). Calibration curves demonstrated acceptable predictive accuracy. Risk stratification based on optimal cutoff (71.099 points) succeeded in differentiating high- and low-risk groups, demonstrating a significant difference in survival in training (HR = 2.53, p<0.001) and validation cohorts (HR = 1.67, p=0.021).

### 3.6 External validation

**Figure 6:**
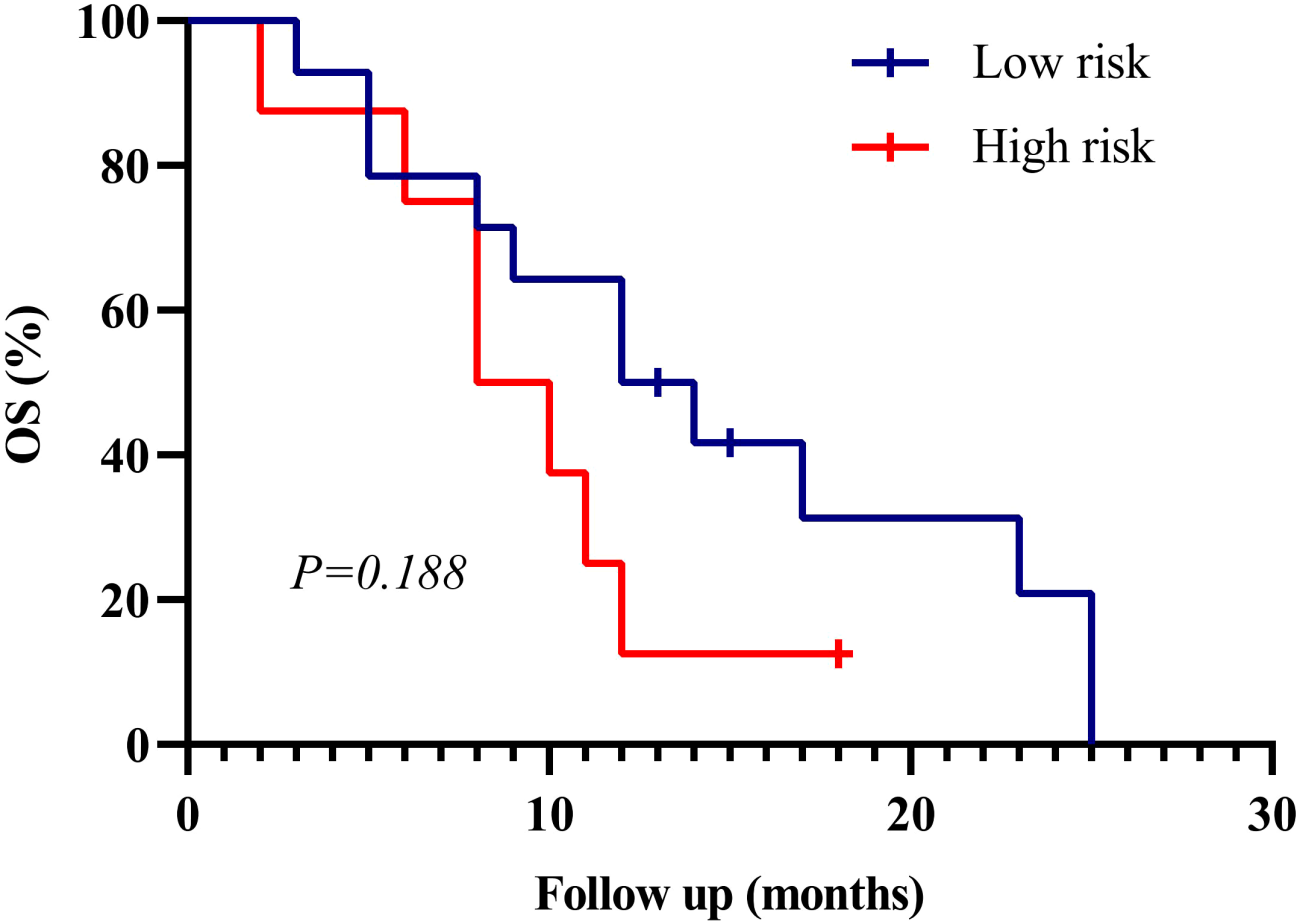
Kaplan–Meier survival curves for external validation cohort (n=22). Patients were stratified into high- and low-risk groups based on the SEER-derived nomogram. Although the difference in overall survival (OS) did not reach statistical significance (*p* = 0.188), a consistent trend toward better prognosis in the low-risk group was observed, supporting the generalizability of the model.

To assess real-world applicability, we applied the nomogram to an independent institutional cohort of 22 DMG patients. Patients were stratified into high- and low-risk groups using the same cutoff value. Survival curves showed a trend toward poorer outcomes in the high-risk group, although the difference did not reach statistical significance (p = 0.188) (**Figure 6**). Baseline clinical features of these patients are summarized in **Supplementary Table 1**.

## 4 Discussion

This population-based study provides an accurate prognosis assessment of the prognosis of DMG, which will reveal important clinicopathological factors influencing survival, and emphasize age-specific patterns of tumor location and therapeutic response, which may help to improve the understanding of DMG and individualized clinical practice.

### 4.1 Age-location dynamics and biological implications

As in previous literature, we found a clear age-dependent tumor site distribution: pediatric DMG was primarily located in the brainstem (classic DIPG); versus adults with mainly thalamus or spinal cord involvement (17–21). Consistent with previous findings, this age-dependent survival difference, combined with the anatomical distribution of tumors, suggesting fundamental biological heterogeneity between pediatric and adult DMGs, with age probably being a key driver for the heterogeneity of the molecular behavior of tumors (17, 19, 22–25). The survival time of children with DMG was significantly different, and the prognosis of the patients with thalamic or spinal cord tumor was better than those with brainstem tumors (26). These observations support the role of developmental biology in disease behavior and advocate for location- and age-adapted treatment strategies. These findings are consistent with prior reports identifying both age and tumor location as independent prognostic factors in DMG, supporting their inclusion in our prognostic model.

### 4.2 Methodological rigor and clinical implications of treatment stratification

One of the main methodological advances in our study was the introduction of a refined three-tiered classification of therapy (radiation therapy alone, chemotherapy alone, or combined chemoradiotherapy) to overcome the limitations of conventional binary classifications (for example, radiation versus no radiation, chemotherapy versus no chemotherapy).Our analysis showed that chemotherapy alone did not provide a survival benefit, while the combination treatment significantly improved the outcome — thus correcting the misleading conclusion that only chemotherapy in the binary analysis improved the prognosis. This difference can be explained by the fact that the brain stem and thalamic areas are particularly intact (BBB), which limits the penetration of chemotherapy agents like temozolomide (TMZ), and eventually reduces the effectiveness of monotherapy (27–29). In addition, the methylation of the MGMT promoter is typically absent in DMG, resulting in a poor efficacy of TMZ (27, 30). On the contrary, it has been found that radiation therapy can temporarily destroy the blood-brain barrier, which may increase the delivery efficiency and therapeutic efficacy of chemotherapy drugs at the tumor site (31). Although SEER lacks molecular data, these interpretations are supported by biological plausibility and prior literature. These findings highlight the need for individualized treatment regimens based on tumor location and biological characteristics, ideally guided by future molecular-integrated datasets.

### 4.3 Surgical resection: limitations and anatomical considerations

Consistent with prior research, our analysis demonstrated that surgical resection extent—biopsy, partial, or subtotal—did not significantly affect OS in DMG patients (32–34). This result highlights DMG’s diffuse infiltrative nature and frequent involvement of anatomically critical midline structures, significantly limiting surgical efficacy. Although our study did not specifically analyze surgical outcomes stratified by tumor location, the predominance of brainstem lesions (where aggressive resection is rarely feasible) likely contributed to this overall negative result.

These findings support minimally invasive biopsy for molecular characterization as the preferred standard approach, given its vital role in diagnosis, prognosis, and eligibility for targeted therapy trials (13). Prospective analyses involving larger cohorts of non-brainstem DMGs may help clarify whether location-specific surgical approaches could provide selective survival benefits.

### 4.4 Clinical application and external validation of the prognostic model

Based on our prognostic nomogram, we propose a risk-adapted management approach to improve clinical outcomes. Patients identified as high-risk may be candidates for early enrollment in clinical trials investigating novel targeted therapies (e.g., ONC201 or GD2 CAR-T therapy), which were not captured in the SEER database but represent promising investigational options for DMG (11, 12, 35–40), while low-risk patients could benefit from standard chemoradiation as recommended by current guidelines. This stratification approach aligns with precision medicine principles, facilitating personalized clinical decisions and optimizing therapeutic strategies. Prospective validation of this strategy is crucial for further refinement of individualized treatment algorithms.

To further evaluate the generalizability of our model, we performed external validation using an independent institutional cohort (n=22). Although statistical significance was not achieved in this small dataset (p=0.081), the direction and magnitude of the risk stratification effect mirrored the SEER findings, suggesting a consistent prognostic trend across populations. Given the inherent challenges in assembling large, histologically confirmed DMG cohorts, this level of validation is rare and valuable. Nonetheless, caution should be exercised in interpreting these results due to the limited sample size, and larger multicenter prospective studies are warranted to substantiate the external validity of our model.

### 4.5 Limitations and future directions

This study is constrained by the inherent limitations of retrospective SEER-based analysis, including the lack of molecular and detailed treatment data. While we incorporated an external validation cohort (n=22) to enhance model credibility, statistical power remains limited. Future prospective studies should incorporate comprehensive molecular profiles (e.g., H3K27M status, MGMT methylation) and multicenter validation to refine prognostic models and strengthen clinical utility.

In addition, socioeconomic factors also emerged as a prognostic variable in our model. Although not directly related to tumor biology, previous studies in glioma and other cancers have shown that lower socioeconomic status is associated with limited access to care, reduced treatment adherence, and worse survival outcomes (41–43). This highlights the need to consider not only biological but also socioeconomic factors in DMG management, and future prospective studies should further examine these associations.

## 5 Conclusions

This study represents the largest SEER-based analysis of DMG to date, establishing a robust prognostic nomogram incorporating demographic, anatomical, and treatment-related factors. The model demonstrated good predictive performance and practical utility in both internal and external validation. Our findings highlight the prognostic value of age, tumor site, and combined therapy, while reaffirming the limited role of extensive resection. Future efforts should prioritize integration of molecular diagnostics and prospective validation, enabling improved risk stratification and personalized care in DMG.

## Data Availability

The original contributions presented in the study are included in the article/**Supplementary Material**. Further inquiries can be directed to the corresponding author.
